# How does early-life adversity affect childhood mental health and cognitive function?

**Published:** 2023-03

**FEBRUARY 8, 2023** - In the study of 13,287 children in the UK, adversity at age 3 years was strongly associated with poorer mental health across all ages from 3 to 14 years. Also, adversity predicted poorer working memory at age 11 and vocabulary at age 14. The impact of adversity on cognition was partially due to its negative effects on mental health during development.

“Our findings not only highlight the deleterious effects of adversity on mental health and cognitive abilities but also reveal one of the mechanisms through which these effects manifest and persist over a long period of time. Prolonged periods of poor mental health as a result of early-life adversity, may have lasting or partially cumulative effects on cognitive abilities of working memory and vocabulary,” said the lead author of the paper, Tochukwu Nweze, PhD, a recent graduate of University of Cambridge. “At a time of rising mental health challenges among teenagers and young people, exacerbated by contemporary environmental risk factors, we suggest that educators in collaboration with clinicians could foster greater resilience by attempting to break this vicious cycle of persistent and self-sustaining mental health difficulties faced by individuals who experienced early adversity through some sort of deliberate and targeted clinical intervention.”

***URL upon publication: https://onlinelibrary.wiley.com/doi/10.1111/jcpp.13757***

***Full citation:** “Childhood mental health difficulties mediate the long-term association between early-life adversity at age 3 and poorer cognitive functioning at ages 11 and 14.” Tochukwu Nweze, Michael Ezenwa, Cyriacus Ajaelu, Chukwuemeka Okoye. Journal of Child Psychology and Psychiatry; Published Online: 08 February 2023 (DOI: 10.1111/jcpp.13757).*

*Copyright © 2019 The Cochrane Collaboration. Published by John Wiley & Sons, Ltd., reproduced with permission.*

